# Fluorescence-Based Rapid Detection of Microbiological Contaminants in Water Samples

**DOI:** 10.1100/2012/234858

**Published:** 2012-04-30

**Authors:** Hervé Meder, Anne Baumstummler, Renaud Chollet, Sophie Barrier, Monika Kukuczka, Frédéric Olivieri, Esther Welterlin, Vincent Beguin, Sébastien Ribault

**Affiliations:** Merck Millipore, Lab Solutions, BioMonitoring, Research & Development, Applications Group, 39, Route industrielle de la Hardt, 67120 Molsheim, France

## Abstract

Microbiological contamination of process waters is a current issue for pharmaceutical industries. Traditional methods require several days to obtain results; therefore, rapid microbiological methods are widely requested to shorten time-to-result. Milliflex Quantum was developed for the rapid detection and enumeration of microorganisms in filterable samples. It combines membrane filtration to universal fluorescent staining of viable microorganisms. This new alternative method was validated using European and United States Pharmacopeia definitions, with sterile water and/or sterile water artificially contaminated with microorganisms. The Milliflex Quantum method was demonstrated to be reliable, robust, specific, accurate, and linear over the whole range of assays following these guidelines. The Milliflex Quantum system was challenged to detect natural contaminants in different types of pharmaceutical purified process waters. Milliflex Quantum was demonstrated to detect accurately contaminants 3- to 7-fold faster than traditional membrane filtration method. The staining procedure is nondestructive allowing downstream identification following a positive result. The Milliflex Quantum offers a fast, sensitive, and robust alternative to the compendial membrane filtration method.

## 1. Introduction

Microbiological contamination of process waters is a key issue for pharmaceutical, biotechnology, and food and beverage industries. Traditional methods are the reference for the control of microbiological quality of water as they are reliable, easy to use and allow microorganisms identification. Nevertheless, these methods are time-consuming and labor-intensive. Moreover, they depend on the ability of microorganisms to yield visible colonies after an incubation period of typically 3 days that can go up to 14 days (European and United States Pharmacopeia). This long time-to-result is a concern for industries as improvement in processes and products requires faster methods to control microbiological quality. Therefore, over the past 25 years, many technologies have been developed to reduce the time-to-result. These new alternative rapid methods have to be sensitive, accurate, and cost-effective. The most studied and used technologies are polymerase chain reaction (PCR), impedimetry, bioluminescence, enzyme-linked immunosorbent assay (ELISA), flow cytometry (FCM), and solid-phase cytometry (SPC) [1–3].

The Milliflex Quantum (Millipore, Molsheim, France) is a new system developed for the rapid detection and enumeration of microorganisms in filterable samples. It combines membrane filtration and fluorescent staining, which are two proven and widely used technologies. The detection is based on a universal enzymatic fluorescent staining of viable microorganisms. The staining procedure is nondestructive allowing downstream identification following a positive result.

This paper describes the validation of the performances of the Milliflex Quantum method. The study was done according to definitions for the validation of a quantitative estimation of viable microorganisms in a sample from European (chapter 5.1.6.) and United States (chapter <1223>) Pharmacopeia. Robustness, ruggedness, accuracy, linearity, range, limit of quantification, limit of detection, and specificity of the method were assessed with sterile water and/or sterile water artificially spiked with microorganisms. The Milliflex Quantum method was also challenged to detect natural contaminants in different types of pharmaceutical process waters. All results obtained with Milliflex Quantum method were compared to the traditional membrane filtration method.

## 2. Materials and Methods

### 2.1. Media

Prefilled Tryptic Soy Agar plates (TSA, Millipore), R2A plates (Millipore), and Sabouraud Dextrose Agar plates (SDA, Millipore) were used to promote growth of microorganisms.

### 2.2. Microorganisms Strains

The following American-Type Culture Collection (ATCC) strains were used to validate the Milliflex Quantum method: *Candida albicans* ATCC 10231, *Aspergillus brasiliensis* ATCC 16404, *Staphylococcus epidermidis* ATCC 12228, *Ralstonia pickettii* ATCC 27511, *Brevundimonas diminuta* ATCC 19146, *Staphylococcus aureus* ATCC 6538, *Pseudomonas aeruginosa* ATCC 9027, *Bacillus subtilis* ATCC 6633, and *Escherichia coli* ATCC 8739. An environmental isolate of *Caulobacter sp.* was also tested with Milliflex Quantum. The cultures were maintained at −80°C in Tryptic Soy Broth (TSB; BioMérieux, Craponne, France) with 5% (v/v) glycerol (Sigma-Aldrich, St. Quentin Fallavier, France) in 0.5 mmol L^−1^ HEPES buffer (Sigma-Aldrich).

### 2.3. Milliflex Quantum Method

The procedure used is as described by Baumstummler et al. [4]. Briefly, samples were filtered through mixed cellulose ester membranes and membranes were placed onto media agar plates and incubated at temperatures recommended by Pharmacopeia or at optimal growth temperatures. After incubation, membranes were stained for 30 min and placed in the Milliflex Quantum reader for counting fluorescent microcolonies. Membranes were reincubated onto media agar plates for visual counting of colony-forming unit (CFU), viability assessment, and contaminants identification. The compendial method was performed in parallel. 

Fluorescence counts and CFU counts obtained after reincubation were compared to the compendial method. The fluorescence recovery and viability recovery were calculated as follows:

(1) $$\begin{array}{cc} & \text{Fluorescence recovery}\;\left(\%\right)\\ & \;=\frac{\text{Fluorescence count}}{\text{Compendial method count}}\times 100,\\ & \text{Viability recovery}\;\left(\%\right)\\ & \;=\frac{\text{CFU count after reincubation}}{\text{Compendial method count}}\times 100. \end{array}$$

Acceptance criterion for these parameters was set to equal to or higher than 70% (European Pharmacopeia chapter 5.1.6. and United States Pharmacopeia chapter <1223>).

### 2.4. Milliflex Quantum Method Validation

#### 2.4.1. Statistical Analysis

An Anderson-Darling test or a Goodness-Of-Fit Chi2-test was used to determine if data obtained with the Milliflex Quantum method and with the compendial method follow a normal distribution. When data are normally distributed (Anderson-Darling *P* value ≥ 0.1; Chi2-value ≤ 4.61 for a 5 classes distribution; Chi2-value ≤ 2.71 for a 4 classes distribution), a one-way analysis of variance (ANOVA), a Student's two samples *t*-test or a Chi2-test was performed to compare results obtained with both methods. All statistical analysis were carried out using the Minitab Statistical Software (version 14; Minitab Inc., State College, PA, USA) except the Goodness-Of-Fit Chi2-test which was performed with Microsoft Office Excel (version 2003; Microsoft, Redmond, WA, USA). 

#### 2.4.2. Negative Controls

Negative controls were carried out in parallel of microorganisms testing. One hundred mL of 0.9% NaCl water (B. Braun Medical, Boulogne Billancourt, France) was filtered, incubated, and analyzed in the same conditions as samples containing microorganisms.

#### 2.4.3. Incubation Time Robustness

Spiked samples were filtered, and membranes were incubated during various times before being stained following the Milliflex Quantum protocol as described previously. Membranes were reincubated to assess viability. Incubation conditions used were those required for the microbiological examination of nonsterile products in chapters of European (2.6.12. and 2.6.13.) and United States (<61> and <62>) Pharmacopeia. Fluorescence and viability recoveries were determined in comparison with the compendial method. An ANOVA was used to assess the time range for stable enumeration with Milliflex Quantum.

#### 2.4.4. Ruggedness

The effect of using different media lots, membrane lots, reagent lots, analysts, and instruments was assessed. *Candida albicans *and* Ralstonia pickettii*, spiked separately in sterile water, were detected using 2 different lots of either membranes or reagents. Moreover, ruggedness of media was evaluated on TSA with *Bacillus subtilis* and *Escherichia coli*, on SDA with *Candida albicans* and on R2A with *Ralstonia pickettii*. For each ruggedness test, two different test runs were performed. Each run was tested by a different analyst, with a different set of instruments. Fluorescence and viability recoveries were calculated, and ANOVA was performed to check if recoveries corresponding to the different tested conditions were not statistically different (*P* value ≥ 0.05).

#### 2.4.5. Accuracy, Linearity, Range, and Limit of Quantification

For each challenged microorganism spiked in sterile water, tests were performed at the following targeted spike levels per sample: 0 CFU, 5 CFU, 25 CFU, 50 CFU, 75 CFU, and 100 CFU. Milliflex Quantum method and traditional method were performed at the same time. Fluorescence and viability recoveries were calculated and a Student's two samples *t*-test was performed to check if the Milliflex Quantum counts were not statistically different from the traditional Milliflex counts (*P* value ≥ 0.05).

Linearity, range, and limit of quantification were established from the data generated during the accuracy test. Acceptance criteria for linearity include an *R* ^2^-value greater than 0.95 (United States Pharmacopeia) and a linear regression slope between 0.8 and 1.2.

#### 2.4.6. Limit of Detection

The microorganisms used for accuracy testing were adjusted separately to approximately 3–5 CFU per 100 mL until at least 50% of the samples showed growth in the compendial method. Twenty replicate samples were assessed with each microorganism with each method. As the aim of the test was to demonstrate that the Milliflex Quantum method enabled to detect 1 CFU, the method had to detect at least one time 1 CFU during the experiment. Furthermore, a Student's two samples *t*-test was performed to check if the Milliflex Quantum counts were not statistically different from the traditional Milliflex counts (*P* value ≥ 0.05). Finally, the equivalence between the Milliflex Quantum proportion of growth and the traditional method proportion was assessed with a Chi2-test (*P* value ≥ 0.05).

#### 2.4.7. Specificity

The specificity of Milliflex Quantum method was established from the data generated during the robustness and accuracy tests, where a panel of microorganisms was tested.

### 2.5. Detection of Microorganisms in Pharmaceutical Process Waters

In-process nonsterile water samples were taken at different steps of the water treatment process in 5 pharmaceutical plants. These various types of purified waters were diluted in 100 mL of 0.9% NaCl water (B. Braun Medical) and filtered. Membranes were placed onto R2A plates (Millipore) and incubated at 32.5°C (European Pharmacopeia General Monographs: Water For Injections; Water Highly Purified; Water Purified). Several incubation times were tested to assess the minimal incubation time required for the fluorescence detection. After incubation, membranes were stained following the Milliflex Quantum protocol. Membranes were reincubated onto R2A plates (Millipore) for visual counting of CFU and contaminants identification. The compendial method was performed in parallel. Fluorescence and viability recoveries were determined in comparison with the compendial method.

## 3. Results

### 3.1. Milliflex Quantum Method Validation

#### 3.1.1. Incubation Time Robustness

**Figure 1:**
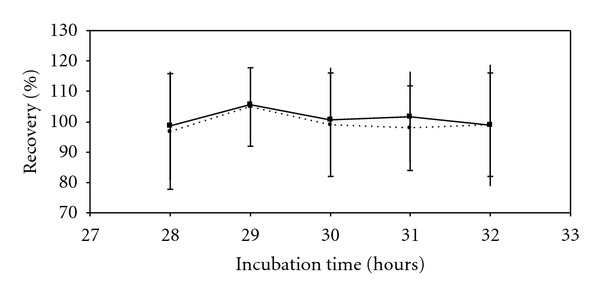
Determination of the robust incubation time range required to detect *Aspergillus brasiliensis* with the Milliflex Quantum method (*n* = 10). Fluorescence recovery (■, solid line); viability recovery (●, dashed line). Standard deviation is denoted by the vertical bars (delineated with—for viability standard deviation).

The robust incubation time range required for detection with Milliflex Quantum was evaluated on 10 microorganisms.  Table 1 summarizes results obtained. Conforming detection of microorganisms was achieved after 22 h of incubation for *Candida albicans* and after 28 h for *Aspergillus brasiliensis*. The minimal incubation time to detect *Escherichia coli* and *Bacillus subtilis* was 8 h and 9 h, respectively. The environmental isolate of *Caulobacter sp.* was detected after 28 hours. The other 5 bacteria tested needed between 12 h and 22 h of incubation. The Milliflex Quantum method was demonstrated to be robust over several incubation times for each strain (fluorescence and viability recoveries ≥ 70% and ANOVA *P* value ≥ 0.05, *n* = 10). These incubation time ranges were used during the further tests of the method validation. Recoveries results over the whole tested incubation time range obtained with *Aspergillus brasiliensis* are presented as example in Figure 1, which proves the results stability and method robustness over the 4-hour tested range.

#### 3.1.2. Ruggedness

Using different media lots, membrane lots and reagent lots has no significant effect on Milliflex Quantum results as ANOVA results proved that fluorescence and viability recoveries were statistically equivalent in all tested conditions (data not shown). Reproducibility of tests results is guaranteed as well with different operators and equipment sets. Therefore, the method's performance is ensured in terms of ruggedness.

#### 3.1.3. Accuracy, Linearity, Range, and Limit of Quantification

*Candida albicans* was accurately detected with the method at each tested contamination level (Table 2) as fluorescence recoveries ranged from 98% to 102% and viability recoveries from 97% to 102%. Moreover, no statistical differences between Milliflex Quantum and traditional method (Student's two samples *t*-test; *P* ≥ 0.05) were found. Similar results were obtained with *Aspergillus brasiliensis, Escherichia coli,* and *Bacillus subtilis* (data not shown).

Linearity, range, and limit of quantification results obtained with microorganisms tested are showed in Table 3. A linear correlation between either fluorescence counts or viability counts and counts obtained with the compendial method were demonstrated since *R* ^2^-values varied from 0.95 to 0.98. The detection observed with Milliflex Quantum ranged from 0 CFU with each microorganism to 97 to 163 CFU, depending on the microorganism tested.

Limits of quantification of the Milliflex Quantum method were demonstrated to be between 4 and 10 CFU, depending on the microorganism tested. These levels are equal to or lower than the corresponding limit of quantification of the compendial method except with *Aspergillus brasiliensis*. With this strain, the limit of quantification of Milliflex Quantum method was found to be 10 CFU, while, 9 CFU was the level demonstrated with compendial method. This latter result is explained by the *Aspergillus* morphology. Due to filaments and colonies merging, the colonies are more difficult to count with the traditional method. The Milliflex Quantum allows earlier counting, with the microcolonies being smaller and separated.

#### 3.1.4. Limit of Detection

The method was proved to detect 1 CFU of *Candida albicans*, *Aspergillus brasiliensis*, *Bacillus subtilis,* and *Escherichia coli* (data not shown). The Milliflex Quantum method gave equivalent result to the traditional method (Student's two samples *t*-test, *P* ≥ 0.05). The Chi2-test demonstrated as well the equivalence of results since proportion of growth is similar for the 2 methods (Chi2-test, *P* ≥ 0.05).

#### 3.1.5. Specificity

The specificity of the Milliflex Quantum method was confirmed as all challenged microorganisms were successfully detected during robustness and accuracy tests.

### 3.2. Detection of Microorganisms in Pharmaceutical Process Waters

Different purified waters were sampled in 5 different pharmaceutical plants, at various stages of the pipes.  Table 4 summarizes the results obtained at incubation times allowing conforming results with Milliflex Quantum and allowing a stable count with the traditional method.

The contaminants of tested pharmaceutical waters were detected with Milliflex Quantum within a time range of 24 to 40 hours. In comparison, 5 to 7 days were needed to visually count all colonies with naked eyes. The Milliflex Quantum detected the contaminants in waters from 3- to 7-fold faster than the compendial method.

After staining and reincubation, the viability rate conformed to acceptance criteria, proving the nondestructiveness of the Milliflex Quantum method. Identifications using the MicroSEQ platform (Applied Biosystems, Carlsbad, CA, USA) were performed on some colonies stained and reincubated. Common water contaminants as *Rhodococcus sp.* and *Delftia acidovorans* and very slow-grower strains as *Aquabacterium parvum* and *Pelomonas saccharophila* were identified, proving that the Milliflex Quantum method is fully compatible with identification technology.

### 3.3. Specific Case Study: Detection of Slow Grower Microorganisms in Pharmaceutical Process Water

In-process nonsterile pharmaceutical water samples were collected after double reverse osmosis, UV and ozone exposure. Microorganisms being highly stressed in these conditions, an incubation time range from 1 to 7 days was applied before detection with Milliflex Quantum and parallel compendial counting were performed until 21 days.

**Figure 2:**
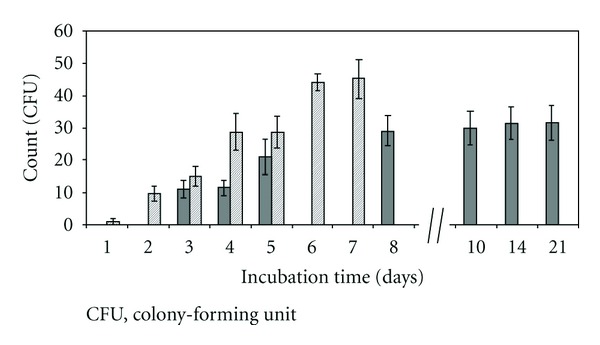
Detection of contaminants in pharmaceutical process water: comparison between fluorescence counts obtained with the Milliflex Quantum method (dashed) and counts obtained with the compendial method (solid grey) (*n* = 5). Standard deviation is denoted by the vertical bars.

**Figure 3:**
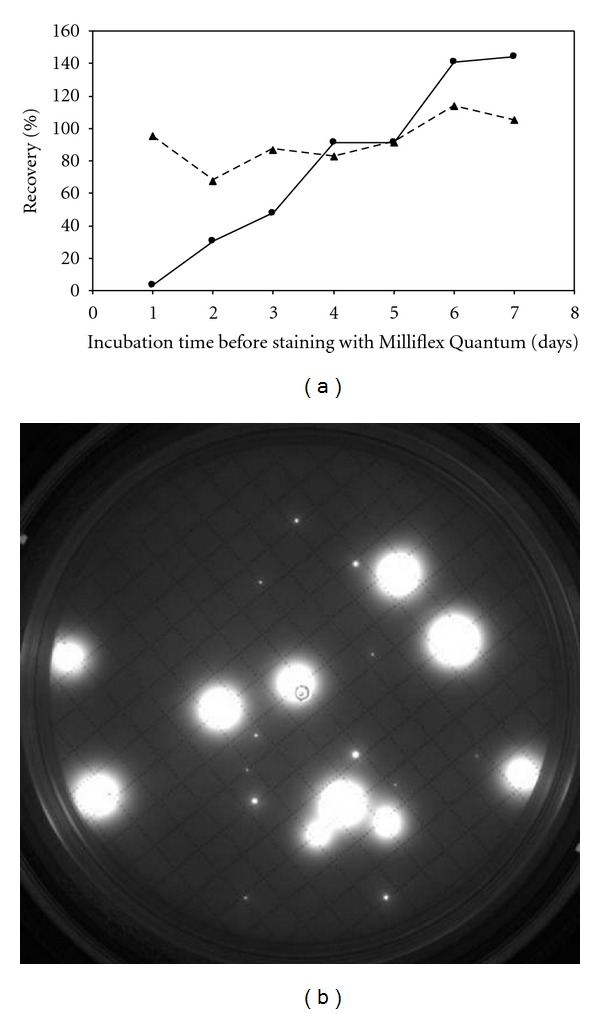
Detection of contaminants in pharmaceutical process water. (a) Fluorescence recovery (●, solid line); viability recovery (▲, dashed line). Viability recoveries were calculated comparing CFU count after a 14-day incubation with both methods. Each viability recovery is placed on the plot with the incubation time before staining with Milliflex Quantum as abscissa value. (b) Example of picture taken after staining of membrane incubated for 4 days on R2A plate at 32.5°C.

Figure 2 compares counts obtained with both methods. With the compendial method, all counts increased up to 14 days of incubation and remained stable between 14 and 21 days. The compendial count at 14 days was chosen as the reference for recoveries calculations. The fluorescence recoveries conformed to acceptance criteria from 4 days of incubation, compared to the compendial count obtained after a 14-day incubation (Figures 3(a) and 3(b)). Viability recoveries were calculated comparing CFU count after a 14-day incubation on membrane stained with Milliflex Quantum and CFU compendial count at 14 days (Figure 3(a)). Conforming viability detection was achieved on membranes stained after 4 days of incubation. The Milliflex Quantum allowed reducing the time-to-results 3.5-fold by detecting accurately contaminants after only 4 days. Identifications by sequencing were performed either on reincubated membranes after staining or on compendial membranes. Fourteen genii were identified: for example, *Variovorax paradoxus*, *Afipia broomeae*, and *Bradyrhizobium japonicum*. The latter was the slowest microorganism present in the samples as almost all colonies becoming visible after the 5th day of incubation or reincubation had the macroscopic aspect of *Bradyrhizobium japonicum*.

## 4. Discussion

We report the validation of the Milliflex Quantum method and the evaluation of its performances for the rapid detection of total microbial contaminants in pharmaceutical water samples. This new rapid method was compared to the traditional membrane filtration method for all tests carried out. The Milliflex Quantum method was demonstrated to be reliable, robust, specific, accurate, and linear over the whole range of assays following these guidelines.

Milliflex Quantum was demonstrated to detect accurately contaminants in 7 pharmaceutical process waters after 24 to 40 hours of incubation, in comparison with 5 to 7 days with the traditional method. An additional sample of highly purified water containing very high stressed microorganisms required 14 days to detect contaminants with the culture-based procedure, whereas Milliflex Quantum enabled to shorten this time-to-result to 4 days. Therefore, Milliflex Quantum allows accurate enumeration of contaminants with time-to-results that are 3- to 7-fold shorter than traditional method. Gram-negative and Gram-positive bacteria, as well as yeasts and molds are universally detected with high sensitivity by the alternative method. Some additional tests proved the time-to-results can be shortened if microorganisms are incubated in optimal growing conditions, following microbiological literature instead of Pharmacopeia guidelines. For instance, *Pseudomonas aeruginosa* and *Staphylococcus aureus* were accurately detected after 9 hours on TSA at 37°C, instead of, respectively, 16 hours and 12 hours at the 32.5°C temperature recommended by the Pharmacopeia, and *Aspergillus brasiliensis* needed 17 hours at 37°C versus 28 hours at 22.5°C (data not shown). Validation of the Milliflex Quantum method, in optimal conditions, as an alternative method for control of microbiological quality is consistent and would enhance time-to-results performances of the Milliflex Quantum system.

The Milliflex Quantum method utilizes mixed cellulose ester membrane filtration for sample preparation, which ensures consistent and reliable results. Large sample volume up to several hundred milliliters can be processed to monitor microbiological quality of very low-contaminated samples. PCR allows testing only small volumes (generally 0.1 to 1 mL) after complex sample preparation methods and FCM available systems are limited to the treatment of a 1 mL volume of sample.

As fluorescent detection is based on metabolism activity of cells, needing integrity of membranes, Milliflex Quantum enables enumeration of all viable-culturable cells, after a short incubation step. Molecular-based methods are unable to distinguish between live and dead cells, which is a shortcoming of methods like PCR [5, 6]. On the other hand, FCM detection needs to be combined with differential staining techniques to enumerate specifically viable cells. Viable-culturable cells final result can only be calculated by comparison with the number of plate count, thus requiring the growth and visualization of colonies on traditional agar plate after one or several days incubation [7].

The method was proved to detect 1 CFU while reaching this sensitivity level with molecular-based method requires development of complex and often laborious sample preparation methods [8]. The SPC's claimed sensitivity is 1 CFU with immediate detection but discrimination is often difficult to distinguish between microorganisms and dust and raw results can only be analyzed by high skilled operator after a long specific training. On the other hand, FCM is not adapted to the detection of rare event and enables direct counting of contamination levels down to 100–200 cells per mL [9, 10]. Moreover, this technology remains complex to be implemented routinely as data analysis is sophisticated and needs experienced operators [1, 11]. Therefore, FCM is perfectly suitable to monitoring of drinking water and wastewater treatments, whereas followup of microbiological quality of pharmaceutical or biopharmaceutical waters remains very limited as they may contain very low numbers of viable microorganisms [12].

The staining procedure was demonstrated to be nondestructive since colonies grow after staining and reincubation and can be collected for identification. Sequencing allowed identifying natural contaminants of tested pharmaceutical waters. Moreover, all standard identifications methods (Gram-staining, biochemical tests, PCR, etc.) were applied successfully on other samples after filtration, staining, and reincubation to prove their compatibility with the Quantum procedure (data not shown). The nondestructiveness of the method is an advantage over molecular-based and adenosine triphosphate-based bioluminescence methods, which do not enable to recover and identify contaminants in case of a positive result.

Implementation and use of new rapid method by industries are limited, among others, by regulatory acceptance [1, 10]. Milliflex Quantum method is a membrane-filtration and growth-based procedure, very close to the principle of the compendial method. Sample preparation and incubation conditions remain identical to traditional microbiology. These features have the advantage to facilitate validation and reassure regulation agencies for their acceptance of rapid method as routine procedure for water monitoring, replacing longer traditional methods.

Milliflex Quantum was demonstrated to be compatible and efficient in beverages and raw materials matrices (data not shown). The method has been also evaluated for its applicability in the detection of low concentrations of microorganisms in animal cell cultures and cell culture media. Combined to a fast pretreatment method to selectively lyse mammalian cells, the time-to-results were 2–5 times shorter than traditional method [13]. This feature is of great interest for monitoring biotechnology processes including high contaminated cells bioreactors.

We demonstrate in this paper that Milliflex Quantum is a validated and reliable tool enabling the rapid detection and enumeration of microbial contamination in pharmaceutical process waters. This easy-to-use protocol and simple system is totally suitable to be implemented in routine for the monitoring of purified water. The Milliflex Quantum system can be applied to a wide range of filterable products as it was proved to be effective as well with beverages, raw materials, and mammalian cells contaminated matrices.

## Figures and Tables

**Table 1:** Determination of the robust incubation time range required to detect collection strains with the Milliflex Quantum method (*n* = 10).

Microorganism	Robust incubation time range	Fluorescence recovery ± SD (%)	ANOVA *P* value on fluorescence recoveries within robust incubation time range	Viability recovery ± SD (%)	ANOVA *P* value on viability recoveries within robust incubation time range
*Candida albicans* ATCC 10231	22 h–26 h	102 ± 38	0.66	101 ± 39	0.75
*Aspergillus brasiliensis *ATCC 16404	28 h–32 h	99 ± 18	0.71	97 ± 19	0.66
*Staphylococcus epidermidis* ATCC 12228	14 h–18 h	98 ± 18	0.20	101 ± 15	0.28
*Ralstonia pickettii *ATCC 27511	22 h–26 h	91 ± 26	0.33	92 ± 26	0.34
*Brevundimonas diminuta *ATCC 19146	22 h–26 h	112 ± 21	0.78	100 ± 18	0.13
*Staphylococcus aureus *ATCC 6538	12 h–16 h	128 ± 33	0.06	117 ± 27	0.33
*Pseudomonas aeruginosa* ATCC 9027	16 h–20 h	111 ± 28	0.91	103 ± 24	0.16
*Bacillus subtilis* ATCC 6633	9 h–10 h	111 ± 23	0.44	102 ± 21	0.40
*Escherichia coli* ATCC 8739	8 h–10 h	122 ± 29	0.06	111 ± 24	0.44
*Caulobacter sp.* (environmental strain)	28 h–32 h	90 ± 17	0.16	102 ± 17	0.10

ANOVA, One-way analysis of variance; ATCC, American Type Culture Collection; SD, Standard deviation.

**Table 2:** Accuracy results obtained with *Candida albicans*: comparison between fluorescence and viability results obtained with the Milliflex Quantum method and compendial method results (*n* = 10).

Microorganism	Incubation time	Target concentration (CFU)	Fluorescence count mean ± SD (CFU)	Fluorescence recovery ± SD (%)	Student's two samples *t*-test *P* value on fluorescence counts	Viability count mean ± SD (CFU)	Viability recovery ± SD (%)	Student's two samples *t*-test *P* value on viability counts
		0	0.0 ± 0.0	NA	NA	0.0 ± 0.0	NA	NA
		5	6.2 ± 2.9	102 ± 61	0.93	6.2 ± 2.9	102 ± 60	0.93
*Candida albicans * ATCC 10231	22 h	25	24.9 ± 5.4	101 ± 29	0.93	24.2 ± 5.6	98 ± 30	0.83
		50	62.7 ± 10.4	98 ± 22	0.73	62.2 ± 10.1	97 ± 21	0.64
		75	102.0 ± 6.3	101 ± 10	0.68	101.4 ± 5.5	101 ± 9	0.82
		100	163.9 ± 15.3	102 ± 15	0.65	161.7 ± 14.7	101 ± 14	0.87

ATCC, American Type Culture Collection; CFU, Colony-forming unit; NA, Not applicable; SD, Standard deviation.

**Table 3:** Linearity, range and limit of quantification results obtained with the Milliflex Quantum method: analysis done using data generated for the accuracy testing of the Milliflex Quantum method, comparison between fluorescence and viability results (*n* = 10).

Microorganism	Linearity				Range (CFU)		Limit of Quantification (CFU)		
	Fluorescence *R* ^2^-value	Fluorescence linear regression slope	Viability *R* ^2^-value	Viability linear regression slope	Fluorescence	Viability	Fluorescence lowest count mean	Viability lowest count mean	Lowest mean count with compendial method
*Candida albican * ATCC 10231	0.98	1.02	0.98	1.01	0 to 163	0 to 161	7	7	7
*Aspergillus brasiliensis * ATCC 16404	0.95	1.09	0.96	1.10	0 to 124	0 to 120	10	10	9
*Bacillus subtilis *ATCC 6633	0.96	1.16	0.96	0.97	0 to 121	0 to 97	6	6	6
*Escherichia coli *ATCC 8739	0.95	1.06	0.96	1.02	0 to 120	0 to 114	4	4	5

ATCC, American Type Culture Collection; CFU, Colony-forming unit.

**Table 4:** Detection of microorganisms in pharmaceutical waters: comparison between fluorescence and viability results obtained with Milliflex Quantum and compendial method results (*n* = 10). “a” and “b” are 2 different types of waters, sampled in the same pharmaceutical plant.

Water	Tested volume	Incubation time	Fluorescence count mean ± SD (CFU)	Fluorescence recovery ± SD (%)	Viability count mean ± SD (CFU)	Viability recovery ± SD (%)	Incubation time	Count mean ± SD (CFU)
1	1 mL	40 h	248.3 ± 31.0	113 ± 19	196.3 ± 21.5	89 ± 14	5 days	248.3 ± 31.0
2	10 mL	24 h	105.4 ± 8.0	110 ± 11	98.8 ± 8.8	103 ± 11	5 days	95.8 ± 6.0
3a	10^−2 ^mL	30 h	21.6 ± 5.0	72 ± 19	22.8 ± 6.4	76 ± 24	7 days	29.9 ± 3.9
3b	10^−2 ^mL	24 h	30.6 ± 2.7	77 ± 15	30.6 ± 1.7	77 ± 14	7 days	39.9 ± 7.1
4a	10^−1 ^mL	24 h	179.7 ± 5.5	95 ± 7	178.3 ± 11.5	95 ± 9	5 days	188.7 ± 12.9
4b	50 mL	30 h	24.0 ± 3.0	74 ± 19	27.7 ± 4.7	86 ± 24	5 days	32.3 ± 7.0
5	1 mL	26 h	21.8 ± 8.5	109 ± 55	17.0 ± 5.1	85 ± 37	5 days	20.0 ± 6.4

CFU, Colony-forming unit; SD, Standard deviation.
